# Iron restriction induces the small-colony variant phenotype in *Staphylococcus aureus*

**DOI:** 10.3389/fmicb.2022.978859

**Published:** 2022-12-08

**Authors:** Shariful Islam, Anna C. Callender, Quynh N. Ho, Catherine A. Wakeman

**Affiliations:** Department of Biological Sciences, Texas Tech University, Lubbock, TX, United States

**Keywords:** *Staphylococcus aureus*, small-colony variant, host-pathogen interface, nutritional immunity, aerobic respiration

## Abstract

Pathogens such as *Staphylococcus aureus* must overcome host-induced selective pressures, including limited iron availability. To cope with the harsh conditions of the host environment, *S. aureus* can adapt its physiology in multiple ways. One of these adaptations is the fermenting small-colony variant (SCV) phenotype, which is known to be inherently tolerant to certain classes of antibiotics and heme toxicity. We hypothesized that SCVs might also behave uniquely in response to iron starvation since one of the major cellular uses of iron is the respiration machinery. In this study, a respiring strain of *S. aureus* and fermenting SCV strains were treated with different concentrations of the iron chelator, 2,2′ dipyridyl (DIP). Our data demonstrate that a major impact of iron starvation in *S. aureus* is the repression of respiration and the induction of the SCV phenotype. We demonstrate that the SCV phenotype transiently induced by iron starvation mimics the aminoglycoside recalcitrance exhibited by genetic SCVs. Furthermore, prolonged growth in iron starvation promotes increased emergence of stable aminoglycoside-resistant SCVs relative to the naturally occurring subpopulation of SCVs within an *S. aureus* community. These findings may have relevance to physiological and evolutionary processes occurring within bacterial populations infecting iron-limited host environments.

## Introduction

*Staphylococcus aureus* is a non-motile, Gram-positive, and facultative anaerobe often observed as microflora on vertebrate skin and residing in the nostrils of almost one-third of the world’s population (Noble et al., 1967; Kuehnert et al., 2006). This pathogen can cause many severe diseases by breaching the hosts’ epithelia, including osteomyelitis, bacteremia, endocarditis, pneumonia, and septic shock (Klevens et al., 2007). Studies have emphasized the pathogen as the second most common agent that causes bloodstream infection and is the most significant cause of bloodstream infection-associated fatalities (Laupland et al., 2013). Invading pathogens, including *S. aureus,* encounter various selective pressures such as the limitation of metal nutrients (e.g., iron, zinc, and manganese) while surviving in host environments (Palmer and Skaar, 2016; Baishya and Wakeman, 2019). Therefore, bacteria adopt several mechanisms to withstand these diverse host environments in response to host-induced selective pressures. The successful outcome of *S. aureus* as a chronic pathogen is contingent on its capability to sense these diverse environments, challenge host immunity, alter its metabolic reactions properly, and acquire or secrete essential nutrients (Krishna and Miller, 2012; Dastgheyb and Otto, 2015; Thammavongsa et al., 2015; Richardson, 2019; Lung et al., 2020). One of the most significant adaptations of *S. aureus* observed in clinical isolates is the development of small-colony variants (SCVs; Proctor et al., 2006, 2014; Bui and Kidd, 2015). Compared to wild-type counterparts, the SCVs of *S. aureus* show some phenotypic differences, such as slower growth, reduced hemolysis, and the lack of carotenoid pigmentation (Proctor et al., 1994). The formation of SCV phenotype provides the *S. aureus* increased tolerance against aminoglycoside antibiotics and heme toxicity and may enable increased intracellular persistence (Wakeman et al., 2012; Tuchscherr et al., 2016; Rollin et al., 2017; Herrin Brittany et al., 2021).

Previous studies have assessed how chemicals present in human hosts can influence the SCV phenotype. For example, one study demonstrated that certain *S. aureus* strains transition to biofilm and SCV-associated lifestyles to survive under the influence of host generated reactive aldehydes (Bui et al., 2015). Another study revealed that various staphylococcal species, such as *S. aureus*, *S. lugdunensis*, and *S. epidermidis*, may produce SCV phenotypes in response to exposures to environmental challenges such as pH 5, 10% NaCl, 4°C, and the penicillin G, and vancomycin (Onyango et al., 2013). The staphylococcal response to reactive oxygen species (ROS) is also frequently studied as these molecules are associated with the host innate immune response. One such study indicated that when *S. aureus* is exposed to sublethal levels of the ROS hydrogen peroxide (H_2_O_2_), there is a distinct, dose-dependent rise in the population of SCVs that are resistant to gentamicin (Painter et al., 2015). Due to its encouragement of adaptive and defensive responses that change cell physiology in ways that also increase antibiotic resistance, stress is a specific environmental factor affecting bacteria’s susceptibility to antibiotics (Poole, 2012). When *P. aeruginosa* biofilms are treated with stressors, including metal ions, antibiotics, and oxidative agents in laboratories, various kinds of colony morphological variants, such as antibiotic-resistant small-colony variants (SCVs) and rugose small-colony variants (RSCVs), are recovered with increasing frequency. This shows that colony morphological variations may be crucial to a biofilm population’s ability to survive when it is subjected to environmental challenges (Boles et al., 2004; Davies et al., 2007; Harrison et al., 2007; Workentine et al., 2010). Metal nutrients, such as Cu and Zn, have a long history of being associated with the emergence of antibiotic resistance in environmental bacteria (Calomiris et al., 1984; Wales and Davies, 2015).

Metal nutrients, such as iron, are essential for most living organisms, including *S. aureus* (Bullen et al., 1999). The pathogenicity of *S. aureus* is significantly reliant upon the iron status of the infected hosts. While defending against pathogens (e.g., *S. aureus*), host immune proteins acquire transition metals such as iron, manganese, and zinc. Therefore, bacteria evolve their strategies to access those metal nutrients to counteract the host-induced defense mechanisms (Lopez and Skaar, 2018). The original hypothesis of this study was that SCVs might also be more resistant to iron restriction since one of the major cellular uses of iron is the respiration machinery that SCVs lack. Therefore, the effect of iron limitation on the respiring wild type and the fermentative SCV *S. aureus* is anticipated to be different in the aerobic condition than the effect in the anaerobic condition. The data herein indicate that one of the major impacts of iron restriction in *S. aureus* is inhibition of respiration and the transient adoption of the SCV phenotype, including the antibiotic recalcitrance characteristic of SCVs. Additionally, prolonged exposure to an iron-starved environment enabled increased emergence of antibiotic-resistant stable SCV subpopulations within an *S. aureus* community.

## Materials and methods

### Chemicals and reagents

2,2ʹ dipyridyl (DIP) was purchased from Sigma Aldrich, United States. All other additional reagents and media were purchased from VWR, USA, and Fisher Scientific, United States.

### Bacterial strains

Newman (Duthie and Lorenz, 1952), a clinical isolate of *S. aureus,* was used for all the experiments and will be referred to as wild type (WT) herein. Additionally, SCV mutant strains were derived from the parental background of the WT Newman isolate. These include the Δ*menB* SCV strain lacking menaquinone production (Wakeman et al., 2012), the Δ*hemB* SCV lacking endogenous heme production (Hammer et al., 2013), and the Δ*cydB*Δ*qoxB* (*cyto*^***−***^) mutant lacking cytochrome oxidases (Hammer et al., 2013).

### 2,2′ dipyridyl treatment in liquid medium

WT and SCV strains were streaked on tryptic soy agar (TSA) plates and incubated at 37°C overnight and for more than 24 h, respectively. Individual colonies from each of the strains were picked in triplicates, then they were grown in tryptic soy broth (TSB) medium in a round bottom 96-well plate and then incubated overnight at 37°C with shaking condition at 150 rpm. Then, the cells were normalized to identical OD_600_ (~0.145). DIP solutions were freshly dissolved in absolute ethanol prior to use for each treatment. Firstly, 1 M DIP stock solution was prepared in absolute ethanol and then diluted to 10 mM DIP by adding TSB. All the appropriate concentrations were prepared in TSB medium in 96-well round bottom plates. Control wells were treated with the exact amounts of ethanol as DIP-treated wells.

Then, 2 μl of normalized cells were added to each well. The ethanol control and the DIP-treated cells were incubated at 37°C on a shaker for 24 h. The 24 h treated samples were homogenized by pipetting up and down before taking the OD_600_ reading on the BioTek plate reader. The data was then graphed and analyzed using one-way ANOVA in GraphPad Prism.

### DIP treatment in solid medium

WT and SCV mutants were streaked on TSA plates and incubated at 37°C overnight and for more than 24 h, respectively. Individual colonies from each of the strains were picked in triplicates, and then they were incubated overnight in a round bottom 96-well plate containing TSB at 37°C in shaking conditions at 150 rpm. Then, the cells were normalized to identical OD_600_ (~0.145). DIP solutions were freshly dissolved in absolute ethanol prior to use for the preparation of all the TSA plates with or without different concentrations of DIP or the exact amounts of ethanol (ethanol control). Firstly, 1 M DIP stock solution was prepared in absolute ethanol and then diluted to 10 mM DIP by adding TSB. Next, warm TSA was poured into 50 ml falcon tubes. The 10 mM DIP and ethanol (with added TSB) were added to the falcon tubes accordingly to make different concentrations of DIP and ethanol control. Then, the medium with different concentrations of DIP and ethanol was poured on the Petri dishes labeled accordingly. The plates were kept at room temperature for one to 2 days to dry, and then each freshly made plate was partitioned (labeled) for all four strains. Cells (2 μl) from the normalized overnight cells were poured and streaked out on the fresh plates (1–2 days old at room temperature). Additionally, 10 μl of normalized cells were added to 90 μl of 1X phosphate buffered saline (1X PBS) and then serially diluted in the 1X PBS. After streaking and pouring of serially diluted cells on DIP-treated and ethanol (no DIP)-treated plates, the plates were incubated at 37°C and observed for the following 3 days. The DIP-treated cells and SCVs could not clearly be observed on 1-day or 2-day post incubation because of their slow growth. However, on the third day, the colonies were clearly visible. Therefore, 3-day post incubation images were chosen for the manuscript.

### Reversion experiment on solid medium

Three-day-old colonies from iron-starved solid medium were streaked on the iron-sufficient TSA plates. The morphology of colonies on iron-sufficient media was observed after 3 days of incubation at 37°C.

### Anaerobic growth condition

The BD GasPak EZ anaerobic system (BD Biosciences, United States) was used to create an anaerobic condition. All the Plates containing the treated and untreated cells were set inside the anaerobic jar containing CO2 gas packs and an indicator to observe the proper maintenance of the anaerobic condition. The anaerobic system containing the plates was incubated inside a 37°C incubator, and the data was recorded after the same time periods as the aerobic conditions.

### Gentamicin treatment

22-h DIP-treated and untreated cells of WT, and *ΔmenB* strains (a representative of SCVs) were normalized to 0.1 OD600. The untreated cells were normalized to iron sufficient medium, whereas the DIP-treated cells were normalized to the same DIP-treated TSB medium. After 4 h of gentamicin (5 μg/ml) treatment in shaking aerobic condition, the cells were diluted and poured on plain TSA plates and incubated at 37°C for ~40 h to observe the CFUs.

### *In vitro* experimental evolution

WT bacteria were streaked on TSA plates and incubated at 37°C overnight. Individual colonies from each of the strains were picked in six replicates, and then they were incubated overnight in 15 ml culture tubes containing 5 ml TSB at 37°C in shaking conditions at 150 rpm. Then, the cells were normalized to identical OD_600_ (~0.2). DIP (1 mM) was prepared in 5 ml TSB medium in 15 ml culture tubes containing 5 ml TSB. Control tubes were treated with the exact amounts of ethanol as DIP-treated wells.

Then, 5 μl of normalized cells were added to the culture tubes containing 1 mM DIP in 5 ml TSB. The ethanol control and the DIP-treated cells were incubated at 37°C on a shaker for 24 h. On every day (until 9 days), 5 μl of cells from DIP treated and control was transferred to fresh medium containing the 1 mM DIP or the same amount of ethanol containing TSB medium in new tubes. Every time, DIP-treated cells were transferred to DIP-Treated fresh medium, and similarly, control cells into the fresh medium with controlled environments. The experiment continued for 9 days. On the 3rd day, the cells were normalized to 0.2 OD_600,_ and 10 μl of normalized cells were serially diluted to observe the number of CFUs/ mL on plain TSA plates for each replicate. Additionally, the normalized (0.2 OD_600_) 1 ml cells were centrifuged and resuspended in 100 μl (900 μl supernatant discarded). The resuspended 100 μl of cells from all the replicated tubes were poured on the 30 μg/ml of gentamicin-containing TSA plates. Sterile glass beads were used to spread the cells on the entire plate. The number of gentamicin-resistant SCV colonies for each replicate was observed after 2 days at a 37°C incubator. Then, the gentamicin-resistant SCV colonies/mL were calculated compared to their corresponding Typical CFUs/mL on plain TSA plates. A similar procedure was followed to measure the gentamicin-resistant SCVs per mL after 9 days. The data was then graphed and analyzed using a one-way ANOVA (Tukey’s multiple comparisons) in GraphPad Prism.

### Cross-streaking for growth rescue of SCVs

Gentamicin-resistant individual isolates that emerged from experimental evolution were cocultured (cross-streaked) with either laboratory-derived *ΔmenB* or *ΔhemB* on solid TSA media to test for increased growth indicative of heme or menaquinone exchange (Hammer et al., 2014). Twenty-four isolates were individually cocultured (cross-streaked) with the *ΔmenB* or *ΔhemB* SCV strains. Growth rescue of the *ΔmenB* SCV but not of the *ΔhemB* SCV by cross-streaking with a mutant indicated that the mutant emerging from was capable of menaquinone production but lacked heme production. Growth rescue of the *ΔhemB* SCV but not the *ΔmenB* SCV by cross-streaking with a mutant indicated the mutant produced heme but not menaquinone.

## Results and discussion

### Iron starvation represses the growth of aerobically respiring *Staphylococcus aureus* to the level of fermenting *Staphylococcus aureus*

Oxygen and iron are important molecules influencing the virulence factors in pathogenic bacteria like *S. aureus* that serve important enzymatic functions and contribute to electron transport system-related reactions. Therefore, an alteration in oxygen and/or iron availability may change the bacterial metabolome (Ledala et al., 2014). Additionally, many studies indicated that the alteration in the availability of oxygen and iron could affect the growth and synthesis of numerous virulent molecules, as well as the antibiotic tolerance of bacteria (Cohen et al., 1967; Dassy and Fournier, 1996; Trivier and Courcol, 1996; Johnson et al., 2008). To assess the impact of iron starvation on WT versus SCV *S. aureus*, we used 2,2′ dipyridyl (DIP) treatment which is a well-characterized iron chelator. It has previously been demonstrated that *S. aureus* SCVs commonly exhibit auxotrophy for hemin or menadione and have faulty respiration (Schaaff et al., 2003; Proctor et al., 2006; Sifri et al., 2006; Dean et al., 2014; Masoud-Landgraf et al., 2016) which results in the *hemB* and *menB* SCV mutants chosen for this work. Additionally, a cytochrome deficient mutant Δ*cydB*Δ*qoxB* (Hammer et al., 2013) was selected as a final control for specifically monitoring the role of aerobic respiration since hemin and menadione have other cellular roles including anaerobic respiration (Wissenbach et al., 1990; Edwards et al., 2020).

**Figure 1:**
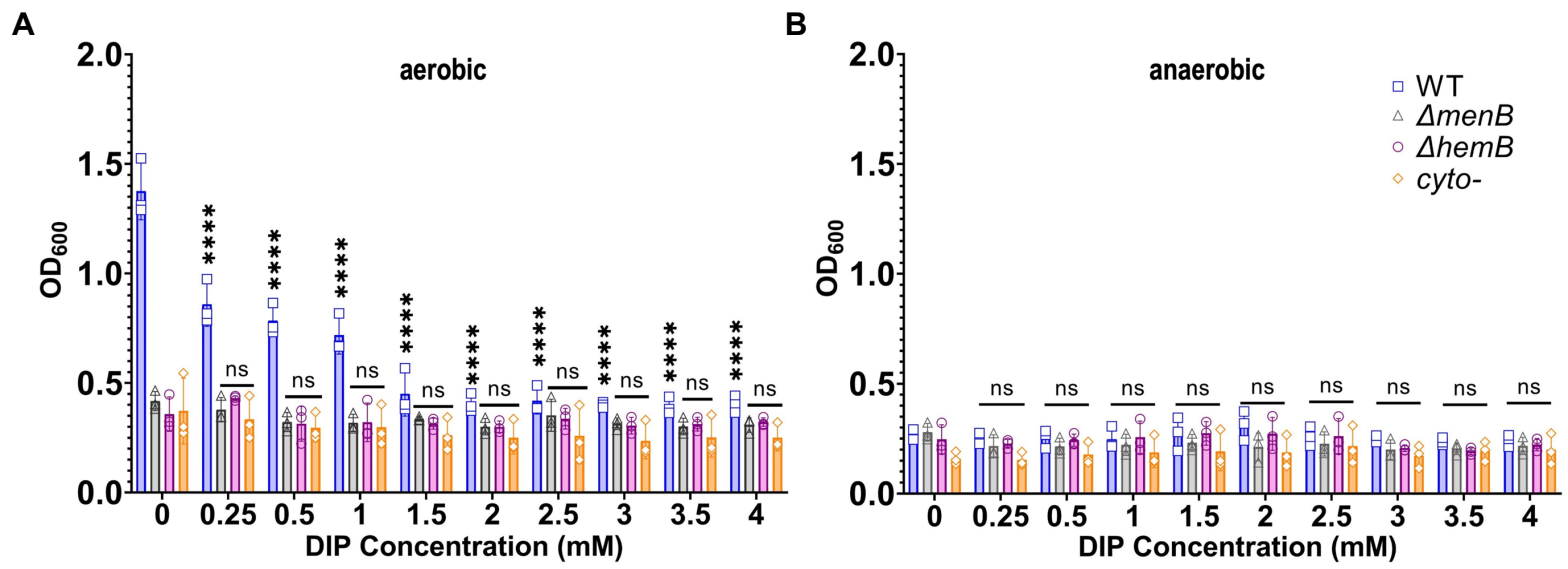
Growth of WT *Staphylococcus aureus* was diminished to the level of SCV growth under iron or oxygen limitation. **(A)** WT and SCV strains were treated with a range of DIP concentrations and cultured aerobically and growth was detected *via* OD_600_ readings. DIP-induced growth suppression is only observed for WT *S. aureus* and only decreases to the level of baseline SCV growth. **(B)** WT and SCV strains were treated with a range of DIP concentrations and cultured anaerobically and growth was detected *via* OD_600_ readings. No DIP-induced growth suppression could be observed for any strain tested. Each of the data points on the graph represents the average of triplicates on each day. Error bars represent standard deviation. A one-way ANOVA (Tukey’s multiple comparisons) was applied to understand the statistical significance of the differences between the control (untreated, 0 mM DIP) and the treatments (“_****_” is the indication for *p* < 0.0001; “NS” is for non-significant data).

Previous studies have often used DIP treatment in the low millimolar range to induce iron starvation in multiple types of microorganisms, including *S. aureus* (Poole et al., 1993; Friedman et al., 2006; Minandri et al., 2016). Therefore, our initial studies used a similar range (0.25-4 mM). Adding DIP caused a significant reduction in the growth rate of respiring WT *S. aureus* but yielded no effect on any of the fermenting SCV strains even at 4 mM DIP as observed by optical density (Figure 1A). The growth suppression of WT was reduced to exactly match the level of SCVs and no further. This finding suggested that a major impact of DIP treatment was to inhibit aerobic respiration. As a further confirmation of this hypothesis, we grew cells under anaerobic conditions. In anaerobic growth, all strains relied on fermentative metabolism and DIP treatment displayed no impact on *S. aureus* growth (Figure 1B).

### Iron starvation causes transient emergence of an SCV phenotype in WT *Staphylococcus aureus*

**Figure 2:**
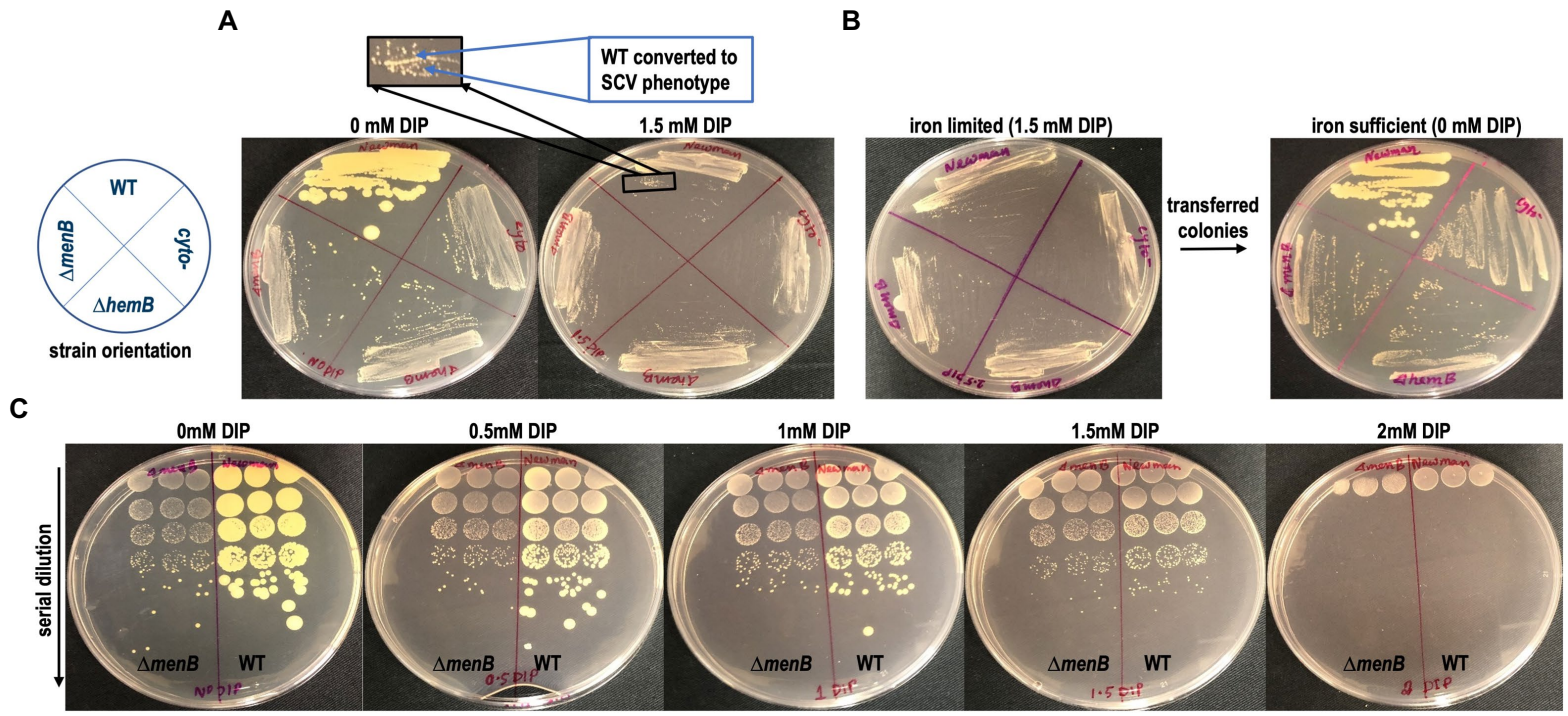
Transient SCV phenotypes are induced in genetically WT *Staphylococcus aureus* during iron restriction. **(A)** WT colonies on TSA are larger and more pigmented than SCV colonies yet are indistinguishable from SCV colonies when grown on TSA supplemented with DIP. **(B)** The SCV conversion is a physiological adaptation to iron limitation rather than a genetic conversion to SCV form as the cells immediately revert to their large, pigmented colony form when subcultured onto iron replete TSA plates. **(C)** The SCV conversion occurs over a large concentration range of DIP treatment and is identical to the SCV conversion induced by anaerobic growth. These plates are representative of data observed on multiple plates cultured on at least 3 separate days.

Because the major effect of iron starvation on *S. aureus* using standard DIP concentrations appeared to be suppression of aerobic respiration, we hypothesized that WT *S. aureus* would exhibit SCV colony morphology when grown on an iron-limited agar plate. Indeed, we observed that genetically WT but iron-starved *S. aureus* adopted a traditional SCV phenotype characterized by a reduction in both colony size and yellow pigmentation (Figure 2A). SCV conversion can be a phenotype induced by the acquisition of a genetic mutant induced by exposure to a stressor (Wilson and Sanders, 1976; Bjorkman et al., 2000; McNamara and Proctor, 2000; Nagaev et al., 2001). Alternatively, SCV conversion could simply be a transient physiological response that will immediately revert when iron levels are restored. To differentiate between these possibilities, we isolated the colonies from an iron-starved plated and restreaked onto iron-sufficient media. Complete and immediate conversion to the normal colony morphology was observed in WT *S. aureus* (Figure 2B).

As none of the SCVs regardless of the genetic origin of the mutation appeared to be differently impacted by DIP treatment, the *ΔmenB* SCV will serve as the SCV control for the remainder of the experiments outlined in this manuscript since menadione auxotrophs are one of the most frequently observed SCV types in the clinic (Proctor et al., 2006). To determine if there were any additional toxicities associated with DIP treatment besides the suppression of respiration, we plated a serial dilution of cells onto DIP-containing plates. At 1.5 mM a complete transition to SCV phenotype can be observed with no reduction in viable cell counts. However, at higher DIP concentrations, other toxicities associated with DIP exposure/iron starvation may be emerging as overall reduction in viable cell counts occur in addition to the SCV phenotypic conversion at 2 mM DIP treatment (Figure 2C).

### Transient SCVs induced by iron starvation exhibit similar antibiotic resistance to genetic SCVs

**Figure 3:**
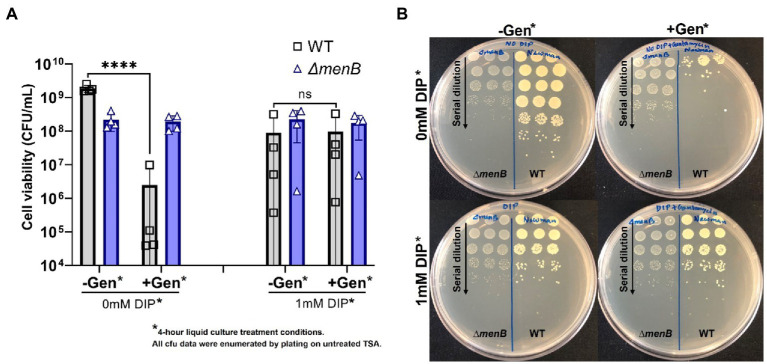
Iron restriction enables WT *S. aureus* to exhibit the aminoglycoside resistance characteristic of the SCV phenotype. **(A)** WT *S. aureus* is highly susceptible to gentamicin treatment but becomes equivalently resistant to this antibiotic as a Δ*menB* strain when iron-restricted with DIP treatment. Each of the data points on the graph represents the average of triplicates on each day and this was repeated for 4 separate days. Error bars represent standard deviation. A one-way ANOVA (Tukey’s multiple comparisons) was applied to understand the statistical significance of the differences (“_****_” is the indication for *p* < 0.0001; “NS” is for non-significant data). **(B)**. The serial dilution plates depict representative CFU data from a single day and were determined by plating onto untreated TSA plates. Treatment conditions listed in the figure represent the conditions from the 4 h incubation period prior to plating on untreated TSA plates for CFU enumeration.

In addition to colony morphology, it was anticipated that the transient SCVs induced by iron starvation might share other fundamental traits with genetic SCVs, including the characteristic antibiotic tolerances. To evaluate this hypothesis, WT *S. aureus* and a representative SCV strain (Δ*menB*) were exposed to gentamicin for 4 h in liquid media in the presence or absence of DIP prior to serial dilution and plating onto untreated media for colony forming unit (CFU) determination (Figure 3). In all conditions, the inherently resistant Δ*menB* strain was unaffected by either iron starvation or antibiotic treatment and grew to equivalent CFUs. WT cells that were not starved of iron were sensitive to gentamicin treatment. On the other hand, when WT cells were transiently iron starved for the 4-h period during the antibiotic exposure, they achieved CFUs similar to the Δ*menB* strain in the presence or absence of gentamicin treatment. Our finding is supported by a previous study that stated that iron starvation induces gentamicin-resistant *Escherichia coli* regulated by small RNA, RyhB (Chareyre et al., 2019).

### Iron starvation enables the emergence of gentamicin-resistant stable SCVs over time

**Figure 4:**
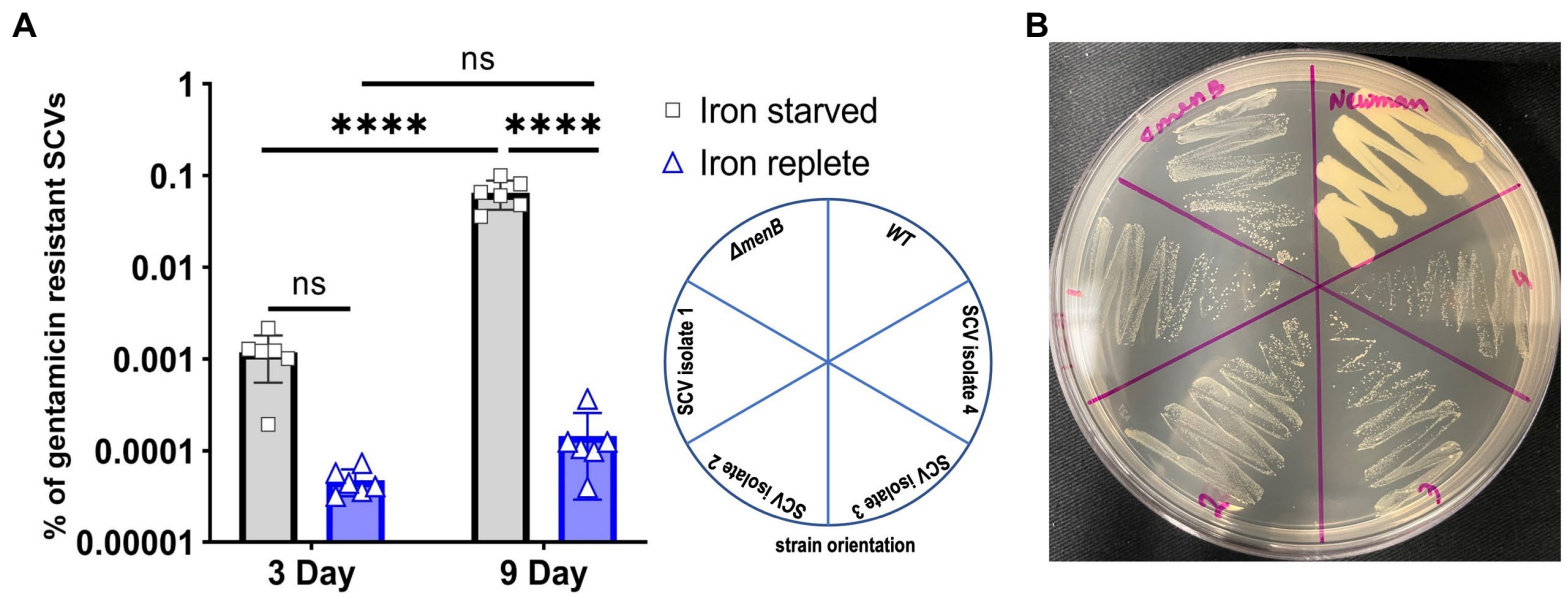
Iron starvation promotes increased rates of stable SCV emergence over time. **(A)** The respiring *Staphylococcus aureus* increased the number of gentamicin-resistant SCVs with the treatment by DIP. Each of the data points on the graph represents one of six replications that were passaged for 9 consecutive days. Error bars indicate the standard deviation. A one-way ANOVA (Tukey’s multiple comparisons) was applied to determine the statistical significance of the differences (“_****_” is the indication for *p* < 0.0001; “NS” is for non-significant data). **(B)** Representative plate assessing the subcultured phenotype of the stable, iron starvation-induced SCV isolates when grown on untreated TSA plates.

We further hypothesized that prolonged growth in iron-starved conditions may enable the emergence of stable gentamicin-resistant SCVs, which has potential clinical implications for chronic infections occurring within iron-resisted host niches. The rationale for this hypothesis is that typically naturally occurring SCV mutations would be selected against within an aerobic *S. aureus* population since respiring *S. aureus* grows more rapidly than fermenting *S. aureus*. However, in iron-starved conditions, our data indicate that there would be no such competitive advantage for the maintenance of respiratory function. This hypothesis was tested using experimental evolution of WT cells passaged in 1 mM DIP for 9 days. The number of gentamicin-resistant colonies was quantified after 3 days and 9 days of DIP treatment (Figure 4A). SCVs were displaying a trend toward increased emergence in iron starvation yet this trend did not achieve statistical significance when analyzed with ANOVA. Of note, the comparison was significant when analyzed by t-test (data not shown). This trend for increased emergence of SCVs under iron starvation appeared more dramatic and did achieve statistical significance by day 9. A previous study indicated that low nutrition combined with a long-time span causes cell heterogeneity in the *S. aureus* population, and stress favors stable SCV cell types (Bui et al., 2015). Therefore, we subcultured several of the gentamicin-resistant SCVs that emerged from the 9-day passaging under iron starvation to assess the phenotypic stability of these populations.

**Figure 5:**
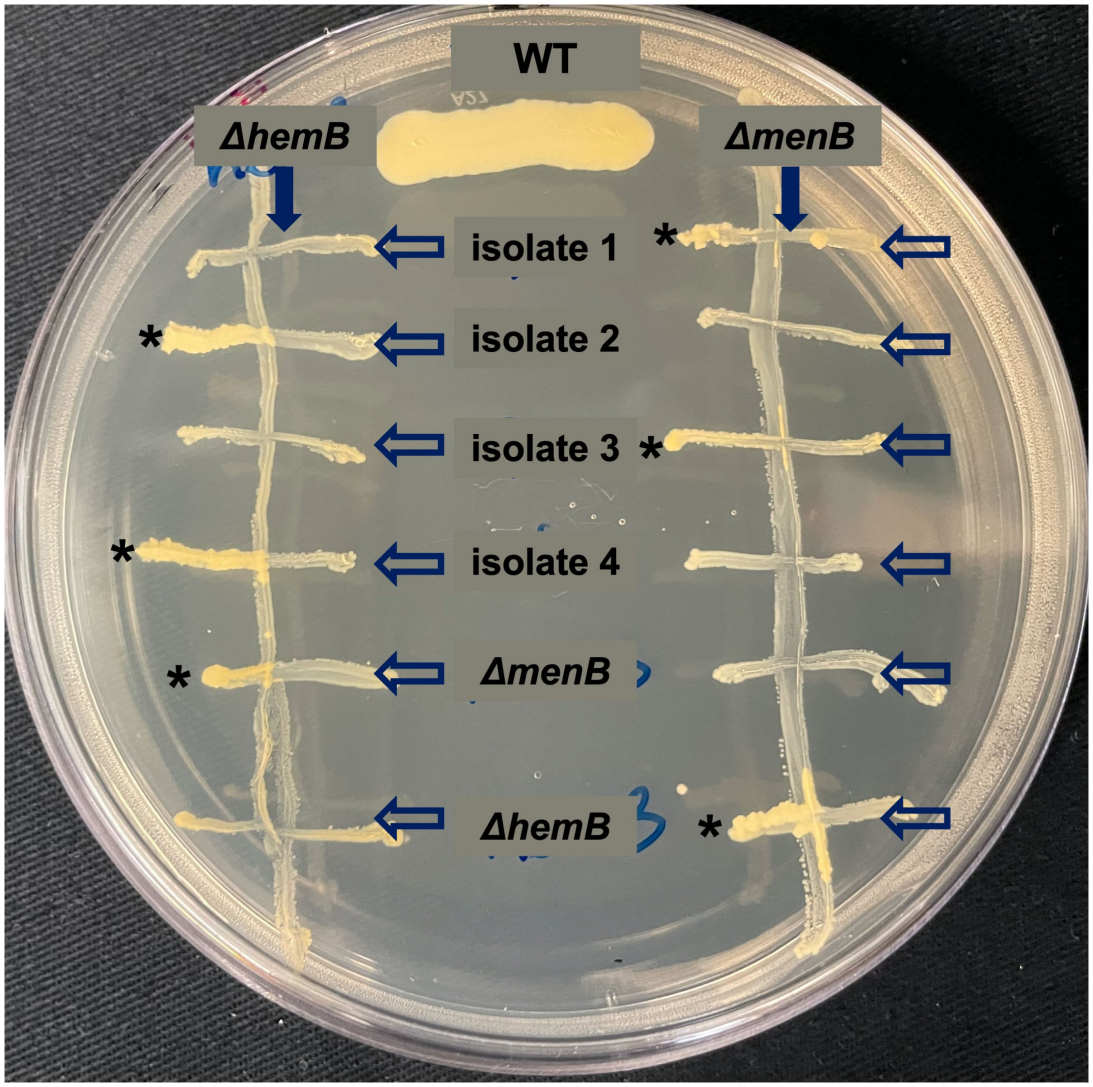
Cross-streaking experiments uncover apparent auxotrophies for stable, iron starvation-induced SCV isolates. Representative plate of cross-streaking experiment in which a menaquinone-deficient *ΔmenB* or a heme-deficient *ΔhemB* were streaked vertically downward as a primary streak (direction of primary streak is emphasized by a solid arrow). SCV isolates and *ΔmenB* and *ΔhemB* as controls were cross-streaked horizontally (direction of the secondary streak is emphasized by unfilled arrows). When a heme-deficient and menaquinone-deficient strain intersect, a moderate growth rescue resembling the WT control streak can be observed (marked with *). In this way, apparent auxotrophies could be assigned for most SCVs that evolved during prolonged exposure during iron starvation. The raw photograph of this plate can be viewed in Supplementary Figure 1.

From each of the six replicated 9-day passaged cultures, four gentamicin-resistant SCVs were selected for further characterization for a total of 24 isolates (herein designated isolates 1–24). When streaked out on untreated TSA, all SCVs that emerged from the passaging experiments exhibited a stable SCV phenotype mimicking that of the *ΔmenB* SCV (Figure 4B). Of the 24 isolates, 17 were stable following multiple subcultures on untreated media whereas 7 exhibited the ability to revert to a WT growth phenotype following repeated subculturing (Table 1). To define an apparent auxotrophy for each isolate, were individually cross-streaked with the laboratory-derived *ΔmenB* or *ΔhemB* SCV strains (Figure 5; Supplementary Figure 1). This type of cross-streaking experiment was previously shown to enable the identification of apparent SCV auxotrophies since *S. aureus* SCV isolates are able to exchange heme and menaquinone to support partial restoration of the respiring phenotype (Hammer et al., 2014). Most of our isolates could be characterized as either menaquinone-deficient strains (9 of 24 isolates) when they achieved growth restoration in the presence of *ΔhemB* but not *ΔmenB* or heme-deficient strains (6 of 24 isolates) when they achieved growth restoration in the presence of *ΔmenB* but not *ΔhemB.* The remaining nine isolates were designated unknown auxotrophies, seven of which were unknown because the SCV phenotype reverted upon further subculture and two of which were unknown because they were not complemented in the presence of either SCV type (Table 1). In total, these data support the contention that brief periods of iron starvation promote a physiological, transient adoption of the SCV phenotype in respiration-capable *S. aureus*, but prolonged exposure to iron starvation can enable the emergence of stable, genetic SCVs within the population.

**Table 1:** Phenotypic assessment of stable, iron starvation-induced SCV isolates.

Isolate #	Apparent SCV auxotrophy	Reversion of phenotype
1	Heme	−
2	Menaquinone	−
3	Heme	−
4	Menaquinone	−
5	Unknown	+
6	Menaquinone	−
7	Menaquinone	−
8	Heme	−
9	Menaquinone	−
10	Unknown	+
11	Unknown	+
12	Menaquinone	−
13	Heme	−
14	Unknown	+
15	Unknown	+
16	Unknown	+
17	Menaquinone	−
18	Menaquinone	−
19	Menaquinone	−
20	Unknown	−
21	Heme	−
22	Unknown	+
23	Unknown	−
24	Heme	−

An isolate was deemed a heme auxotroph if cross-streaking with the heme-producing, menaquinone-deficient Δ*menB* SCV resulted in a growth rescue. An isolate was deemed a menaquinone auxotroph if cross-streaking with a menaquinone-producing, heme-deficient Δ*hemB* SCV resulted in a growth rescue. Unknown auxotrophy indicates that either the isolate did not experience a growth rescue with either cross-streak or they were not a stable SCV that could be subjected to cross-streaking. An isolate in which the SCV phenotype reverted to normal growth upon passaging to fresh media is marked in with a “+” whereas a stable, non-revertible isolate is marked with “−”.

## Conclusion

The findings presented herein indicate that a major consequence of iron starvation in *S. aureus* is the inhibition of aerobic respiration. Additionally, we observe that the transient SCVs induced as a physiological response to iron starvation are equivalently resistant to aminoglycoside exposure as compared to genetic SCVs. Because certain niches of the host environment are known to be iron restricted *via* various mechanisms of nutritional immunity (Palmer and Skaar, 2016), these findings indicate that many *S. aureus* infections may contain inherently antibiotic-resistant cells even when SCVs are not found to be present during clinical laboratory investigation. Furthermore, experimental evolution demonstrated that SCV cells can emerge naturally within an *S. aureus* population at greater rates when cells are cultured under iron restriction. This finding could have implications during infection in that *S. aureus* chronically infecting iron-restricted host niches might become dominated by naturally occurring genetic SCVs without prior antibiotic exposure.

## Data availability statement

The original contributions presented in the study are included in the article/Supplementary material, further inquiries can be directed to the corresponding author.

## Author contributions

SI contributed to experimental design, data acquisition, data analysis, and manuscript drafting. AC and QH contributed to data acquisition and manuscript editing. CW contributed to project and experimental design, data analysis and interpretation, and manuscript drafting and editing. All authors contributed to the article and approved the submitted version.

## Funding

Research in the Wakeman lab was supported by NIH/NIGMS (R15GM128072). Additionally, SI was awarded publication funding from Texas Tech American Society for Microbiology, received a long summer research assistantship from the Department of Biological Sciences at Texas Tech University, and was awarded the Doctoral Dissertation Completion Fellowship (DDCF) from Texas Tech Graduate School. AC received support from the Texas Tech University Centre for the Integration of STEM Education and Research (CISER).

## Conflict of interest

The authors declare that the research was conducted in the absence of any commercial or financial relationships that could be construed as a potential conflict of interest.

## Publisher’s note

All claims expressed in this article are solely those of the authors and do not necessarily represent those of their affiliated organizations, or those of the publisher, the editors and the reviewers. Any product that may be evaluated in this article, or claim that may be made by its manufacturer, is not guaranteed or endorsed by the publisher.
