# Supplementary figures and images for: Iron restriction induces the small-colony variant phenotype in Staphylococcus aureus
Source: Front Microbiol. 2022 Dec 8;13:978859. doi: 10.3389/fmicb.2022.978859 (PMC9772265; doi:10.3389/fmicb.2022.978859)

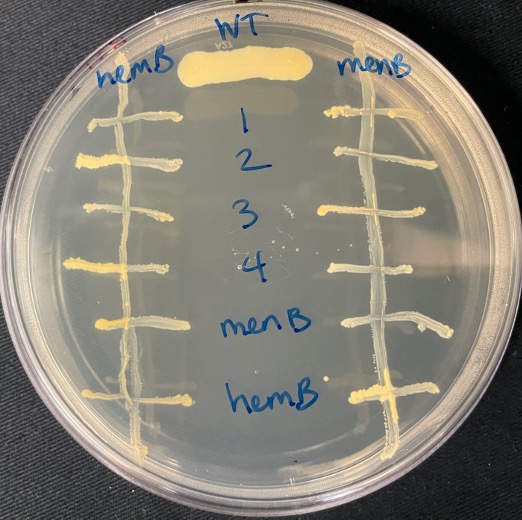

Supplement: Supplementary file 1 [file Image_1.JPEG]
